# Abstracts

**DOI:** 10.1155/S1463924687000130

**Published:** 1987


Journal of Automatic Chemistry/*Journal of Clinical Laboratory Automation*, Vol. 9, No. 2 (April–June 1987), pp. 57–58

### Abstracts

### Page 59

### An evaluation of the EPOS selective chemistry analyser

G. J. Hughes and D. J. Wright

The EPOS (Eppendorf Patient Oriented System) selective chemistry analyser was evaluated using three chemistries: calcium, urea and gamma-glutamyl transpeptidase (GGT).

The dispensing system, photometer unit and temperature control system were all found to be excellent and complied with the manufacturers stated specifications. The between-day and within-batch precisions were acceptable for all three chemistries as was the sample to sample carry-over. The linearity of the urea and GGT methods was shown to extend well above the reference ranges for each and the calcium method was linear between 1·5 mmol/l and 3·3 mmol/l. The urea and GGT methods correlated very well with comparative methods when they were used to measure patient samples and they showed reasonable accuracy when used to measure reference sera although both had a small positive bias. The calcium method, however, showed a very poor correlation against a comparative method and the measurement of reference sera showed a positive bias at high values and a negative bias at low values which may have been related to a calibration problem.

Overall, the instrument was found to be well designed and simple to use. An assessment of safety was satisfactory and running costs were found to be low.

### Page 66

### Real-time spectral evaluation for gas chromatography — Fourier transform infra-red spectrometry

Robert L. White

A GC/FT-IR data collection/evaluation system is described which can be used to rapidly acquire GC/FT-IR infra-red data while simultaneously evaluating previously collected mixture component spectra. Hardware consists of dual microprocessors with parallel processing and multitasking capabilities. Data evaluation is accomplished using low priority macro programs. Data evaluation procedures can be modified by changing program references in a general-purpose macro program. Performance of the GC/FT-IR data collection system is demonstrated for qualitative analysis using library search and for quantitative analysis of methanol and ethanol in complex mixtures.

### Page 72

### A method for determining total nitrogen in Kjeldahl digestion solution using a centrifugal analyser

J. W. Geiger *et al.*

A method has been developed for analysing Kjeldahl digestion solutions utilizing a centrifugal analyser. Nutritional assessment programs generally rely on a urinary urea nitrogen determination to evaluate the protein status of patients. Other protein losses are not evaluated using the urea nitrogen approach, leading to a limited nitrogen evaluation. This method makes is possible to determine total nitrogen in physiological solutions (urine, stools, drainages) in a hospital setting for the purpose of determining protein status in nutritionally depleted patients. Centrifugal analysers are available in many hospital laboratories. Classical Kjeldahl digesters are not convenient to use in a clinical setting; however, the recent development of small self-contained digestion blocks have made the digestion techniques acceptable in any laboratory. After one hour of digestion, the sample is colorimetrically analysed. Monitoring protein status in these patients provides needed information to the Nutritional Assessment Team, which is responsible for recommending appropriate levels of nutritional intake. Recovery of nitrogen added to a urine sample ranged from 97·3% to 100·6%. Coefficients of variation were less than 4%. This method is easily adaptable to most hospital laboratories.

### Page 77

### An evaluation of the Eppendorf EPOS 5060 biochemistry autoanalyser

F. Antoja *et al.*

An evaluation has been made of the EPOS 5060, a selective automatic analyser from Eppendorf, according to the Spanish Society for Clinical Chemistry's guidelines. The system's practicability has been studied, and the analytical units in terms of routine working, covering inaccuracy, imprecision, linearity and photometric drift have been evaluated. The sampler and the reagent dispensing system have been checked for inaccuracy and temperature control.

A rise of photometric imprecision has been observed with decreasing absorbance; linearity is good, drift is negligible and the imprecision of the pipette delivery system is acceptable.

Imprecision (within-run and between-run), carry-over and relative inaccuracy have been studied under routine working conditions for several procedures: creatinine, total protein, ASAT, glucose and alkaline phosphatase. Carry-over was not detected, imprecision was low for enzyme activity measurement and acceptable for the rest of the constituents.

Relative inaccuracy was acceptable except for total protein, when compared with the measurements on the Ultrolab-Aurora. The protein problem could be caused by a calibration.

### Page 82

### The effect of instrumental and environmental factors on the thermal regulation of the temperature of incubation

C. A. Burtis, International Federation of Clinical Chemistry

The thermal regulation of a reaction cuvette and its contents entails the dynamic balancing of the opposing actions of heat input and loss. For this process to occur in a predictable and reproducible manner, heat fluxes must be controlled where possible and heat contributions from unregulated sources must be minimized. In practice, this latter situation is ensured by controlling the ambient temperature of the environment or localized heating and cooling within the instrument.

Since modern-day instruments are designed to operate world-wide in a variety of environments, the EPI recommends that future instruments be designed to provide both regulated heating and cooling capabilities. Improved temperature control should result, and the effect of ambient temperature will be minimized.

---

### Page 87

### The use of queueing theory for planning automated analytical systems

T. L. Pap and L. Leisztner

Queueing theory is suitable for planning laboratories which need to analyse large series of samples. When the distribution of service time and inter-arrival time are not independent, then only a numerical solution to the problem is possible.

In this paper, the authors describe an algorithm, which provides an appropriately simplified model of the laboratory. This algorithm is very useful for calculation of the queueing parameters. The function of the analytical system was modelled and the statistical data were processed by the help of computer program written on the basis of the algorithm. An analytical system suitable for processing 50 000 samples in a year was designed based on the results obtained.

---

### Page 92

### Automated, quantitative, low-pressure, cation-exchange chromatography of haemoglobin variants on midget columns

R. S. Ersser *et al.*

A computer-controlled prototype system for automated, quantitative, liquid chromatographic separation of mixtures of human haemoglobins is described. Cation-exchange chromatography was performed on a 5 × 4·5 mm midget column, filled with 10 μm diameter speres of porous (300A) silica, coated with polyaspartic acid. The column was eluted, using a low-pressure peristaltic pump, with gradients of sodium ions in a bis-tris buffer (40 mM, pH 6·2).

Gradient profiles suitable for general haemoglobin studies (27 min cycle), glycated haemoglobin (20 min cycle) and neonatal screening for sickle-cell disease (10·5 min cycle) have been devised. The precision and accuracy of these methods was investigated, using both reference materials and samples from patients with haemoglobinopathies assayed by a variety of alternative methods. Peak elution times, between batch, had a coefficient of variation (CV) less than 0·8%, peak area versus amount of haemoglobin loaded was linear between 30–350 μg, and the percentage total of haemoglobins, between batch, had CVs less than 5%.

---

### Page 97

### Performance characteristics of a light-scattering immunoassay for thyroxine on a discretionary analyser

Roy Jaggon and Christopher P. Price

The development of an immune complex by reaction between an antigen and antibody can be monitored by nephelometry or turbidimetry. The technique is used for the quantitation of specific proteins. The sensitivity of a method can be enhanced by using an antibody bound to a light-scattering particle. This approach can only be used directly for the measurement of polyvalent antigens.

Hapten molecules can be coupled to a core molecule such as a particle or a large protein molecule; this effectively produces a polyvalent antigen characterized by the hapten. If this reagent reacts with specific antibody to the hapten an immune complex is formed; formation of the complex may be inhibited in introducing a sample containing the hapten.

This paper describes experience with a light-scattering inhibition assay for the measurement of thyroxine.

---

### Page 100

### Comparative precision of laboratory methods

K. F. Yee

The F-test is commonly used to statistically compare the precision of two laboratory quantitation methods. However, when the same samples are used between methods, the F-test is inappropriate and could fail to detect the superior method. The appropriate statistical test in such a situation is the Pitman's test which was first published in 1939.

